# Environmental factors influence the *Haloferax volcanii* S-layer protein structure

**DOI:** 10.1371/journal.pone.0216863

**Published:** 2019-05-10

**Authors:** Thiago Rodrigues-Oliveira, Amanda Araújo Souza, Ricardo Kruger, Bernhard Schuster, Sonia Maria de Freitas, Cynthia Maria Kyaw

**Affiliations:** 1 Department of Cell Biology, Institute of Biological Sciences, University of Brasília, Brasília, Brazil; 2 Department of NanoBiotechnology, Institute for Synthetic Bioarchitectures, University of Natural Resources and Life Sciences, Vienna, Austria; Universitetet i Bergen, NORWAY

## Abstract

S-layers commonly cover archaeal cell envelopes and are composed of proteins that self-assemble into a paracrystalline surface structure. Despite their detection in almost all archaea, there are few reports investigating the structural properties of these proteins, with no reports exploring this topic for halophilic S-layers. The objective of the present study was to investigate the secondary and tertiary organization of the *Haloferax volcanii* S-layer protein. Such investigations were performed using circular dichroism, fluorescence spectroscopy, dynamic light scattering and transmission electron microscopy. The protein secondary structure is centered on β-sheets and is affected by environmental pH, with higher disorder in more alkaline conditions. The pH can also affect the protein’s tertiary structure, with higher tryptophan side-chain exposure to the medium under the same conditions. The concentrations of Na, Mg and Ca ions in the environment also affect the protein structures, with small changes in α-helix and β-sheet content, as well as changes in tryptophan side chain exposure. These changes in turn influence the protein’s functional properties, with cell envelope preparations revealing striking differences when in different salt conditions. Thermal denaturation assays revealed that the protein is stable. It has been reported that the S-layer protein *N*-glycosylation process is affected by external factors and the present study indicates for the first time changes in the protein structure.

## 1. Introduction

Haloarchaea are able to thrive in hyper saline environments such as salt lakes, the Dead Sea, natural brines and marine solar salterns [1]. Indeed, some of these microbes exhibit optimal growth at salt concentrations approaching the saturation point [2]. In order to survive in such conditions, they present several adaptations to maintain their cellular proteins stable and active [3]. Haloarchaea have a high intracellular content of potassium ions to counterbalance the high sodium concentrations in the environment [4, 5]. Proteins also tend to be rich in surface-exposed negatively charged amino acids [6–8], improving solubility at high salt concentrations [9]. Furthermore, all conserved haloarchaeal proteins described hitherto exhibit an acidic nature [10].

Among the most studied haloarchaea, special attention has been given to *Haloferax volcanii*, a moderate halophile frequently used as a model organism for this domain [11, 12]. First isolated from deep sediments of the Dead Sea [13], optimal growth occurs at 1.7–2.5 M NaCl, 45°C, and at slightly acidic pH values [14]. Like many archaea, the *H*. *volcanii* cell envelope consists of a highly ordered protein surface layer, known as the S-layer, anchored directly to the cell membrane [15]. S-layers are currently known to be involved in surface recognition and cell shape maintenance, as well as functioning as protective coats, molecular sieves and molecule and ion traps [16]. Considering that these proteins are produced in high amounts within the cell, often constituting the only cell wall component in archaea, they represent a significant portion (10–15%) of the organism’s total protein content [16–19].

S-layers are composed of one or, in a few cases, two different proteins that self-assemble into stable two-dimensional symmetric lattices [20]. Different S-layer lattice type symmetries are known to exist, with that found on the *H*. *volcanii* cell surface consisting of monomer repeats of six proteins arranged in a hexagonal fashion [13]. These acidic proteins (pI 3.44) form 12.5 nm high complexes, with a 4.5 nm dome-shaped domain at the tip, a 6.0 nm glycosylated spacer element and a 2.0 nm globular domain near the cell surface [21]. Furthermore, the *H*. *volcanii* S-layer protein theoretical molecular weight is of approximately 85.2 kDa and its primary structure consists of 827 amino acids, with seven potential *N*-glycosylation sites. *O*-glycosylation is also known to occur, especially on Thr residues close to the C-terminus portion of the protein. Interestingly, it has been shown that NaCl and divalent cations affect the structural stability of this haloarchaeon’s cell envelope [22].

The first detailed description of a prokaryotic glycoprotein was that of the S-layer protein of the extreme halophilic archaeon *Halobacterium salinarum* [23]. Because of this landmark, halophilic S-layer proteins have attracted significant interest and have been frequently used as study models for post translational modifications in *Archaea* [24–26]. This has led to a considerable number of studies investigating such topics for the *H*. *volcanii* S-layer, with the *N*-glycosylation process for the protein playing an important role in maintaining cell envelope stability and cell viability in hypersaline environments [27]. The protein is also lipid modified by a derivate of mevalonic acid [28], with these modifications depending on the protein’s C-terminus removal by an archaeosortase (ArtA) [26].

Despite the common features and functions shared among different archaeal groups, the similarity between S-layer protein nucleotide and/or amino acid sequences is generally low. Furthermore, a search for archaeal S-layer protein folding models in the Protein Data Bank (RCSB PDB) shows that there are only entries for *Methanosarcina* spp. (PDB code 3U2H and 1L0Q) [29, 30]. Thus, there are still several structural aspects to be explored for archaeal S-layer proteins. While there are studies addressing the *H*. *volcanii* S-layer protein’s primary structure and post-translational modifications [24–26, 31], to date there have been no reports investigating the protein’s secondary and tertiary structures, or describing the behavior of the protein under different solvent conditions. It is worth pointing out that the understanding of structural aspects of archaeal S-layer proteins is of utmost importance in elucidating the molecular mechanisms involved in the evolution and self-assembly properties of this intriguing and complex cell surface component. Furthermore, considering that S-layers have been extensively demonstrated as being suitable to different biotechnological applications [19, 32–35] and haloarchaeal S-layer proteins have yet to be used for such purposes, this structural knowledge could provide data concerning their applicability. Given this, we analyzed the secondary and tertiary organization of *H*. *volcanii* S-layer proteins, as well as the roles of pH, temperature and salt concentrations on the structure and self-assembly properties of the protein.

## 2. Materials and methods

### 2.1 Cell growth conditions

*Haloferax volcanii* DS2 lyophilized cells were kindly provided by the Fundação Oswaldo Cruz’s (FIOCRUZ) culture bank. Cells were grown in *Halobacterium* medium (ATCC 974) at 37°C under agitation, for different time periods depending on the experiment to be performed with periodic transfers to fresh media.

### 2.2 S-layer proteins extraction

*H*. *volcanii* cells were grown to late exponential phase. Cells were submitted to S-layer protein extraction procedures as described by Sumper *et al*., 1990 [31], where cells were treated with EDTA to remove the proteins, resulting in spheroplasts. The resulting protein profile was analyzed by SDS-PAGE [36] and quantified using a Quick Start Bradford Protein Assay (Bio-Rad) kit.

### 2.3. Cell envelope preparations

*H*. *volcanii* cells were grown to late exponential phase and cell envelope preparations conducted according to protocols described by Kessel *et al*., 1988 [21], with minor modifications. Briefly, cells were centrifuged and pellet resuspended in a 2.14 M NaCl and 0.25 M MgCl_2_ salt solution. Cell suspensions were then frozen in liquid nitrogen. Cells were then thawed at room temperature, incubated with DNase (10μg/mL) for one hour at 37°C and cell suspensions centrifuged for 30 seconds at 14.000 *x g* to remove unbroken cells and debris. Supernatant was centrifuged again for 7 minutes under the same conditions. The resulting pellet was resuspended in different salt solutions (0.001 M CaCl_2_; 0.01 M CaCl_2_; 2.14 M NaCl and 0.01 M CaCl_2_; 2.14 M NaCl and 0.25 M MgCl_2_; 0.25 M MgCl_2_) previously employed in studies evaluating salinity influence on the *H*. *volcanii* cell envelope [22].

### 2.4. Transmission electron microscopy

Cell envelope preparations were deposited on pioloform coated copper grids for 1 minute and then fixed using a 2.50% glutaraldehyde solution. Negative staining was performed by immersing grids in a 1% uranyl acetate solution for 45 seconds. Samples were analyzed in a FEI Tecnai G2 20 electron microscope operating at 160 kV.

### 2.5. Secondary structure analyses through circular dichroism

Circular dichroism was performed on a Jasco J-815 (Jasco Corporation, Tokyo, Japan) spectropolarimeter equipped with a Peltier temperature control system (Analytical Instruments, Japan). The Far-UV CD spectra (190–260 nm) were recorded at 25°C, using 0.32 mg/mL of *H*. *volcanii* purified S-layer proteins, in a 0.1 cm cuvette. The assays were performed employing 2 mM sodium acetate, pH 4.0, and 2 mM Tris-HCl, pH 7.0 and 8.5. Additionally, the Far-UV CD spectra were also obtained as a function of different salt concentrations (0.001 M CaCl_2_; 0.01 M CaCl_2_; 2.14 M NaCl and 0.01 M CaCl_2_; 2.14 M NaCl and 0.25 M MgCl_2_; 0.25 M MgCl_2_) at pH 6.8, using 0.13 mg/mL of *H*. *volcanii* purified S-Layer proteins. Twenty successive scans were accumulated and the mean spectrum was recorded using a scanning rate of 100 nm/min and response time of 1 sec to bandwidth of 1.71. The CD signal contribution of the buffer was subtracted from each spectrum. The ellipticity values were converted into molar ellipticity ([θ]) (deg.cm^2^.dmol^-1^) based on a mean molecular mass per residue of 115 Da. The secondary structure content as a function of temperature, pH and salt effects were estimated using the BeStSel platform [37].

Thermo stability curves were obtained at 208 nm, at pHs 4.0 and 7.0, with temperature increasing from 25 to 95°C at a scan rate of 0.2°C/min. Simultaneously, CD spectra in the Far-UV region at 10°C intervals with data pitch of 0.2 nm were also registered. Thermal denaturation curves were generated plotting molar ellipticity ([θ]) at 208 nm against temperatures ranging from 25 to 95°C [38].

### 2.6. Tertiary structure analyses through fluorescence spectroscopy

Fluorescence measurements were performed in a Jasco FP-650 spectrofluorimeter (Jasco Corporation, Tokyo, Japan) equipped with a Peltier temperature control system (Analytical Instruments, Japan). A protein concentration of 0.032 mg/mL was used in assays evaluating the effect of pH on the protein tertiary structure, with 10 mM sodium acetate, pH 3.5–5.5, and 10 mM Tris-HCl, pH 6.0–9.0, employed as buffers. Assays for evaluation of the influence of salt concentrations on the protein were performed using 0.043 mg/mL of purified *H*. *volcanii* S-layer proteins. All salt solutions were as described in circular dichroism assays. Emission spectra were obtained over a 300–400 nm range, at 25°C, with tryptophan excitation at 295 nm and slits of excitation and emission set to 5 nm.

### 2.7. Dynamic light scattering (DLS)

DLS measurements were performed on a Malvern Zetasizer Nano (Malvern Instruments Limited, Worcestershire, United Kingdom) using 0.320 mg/mL of *H*. *volcanii* purified S-layer protein samples. Assays were performed as function of temperatures ranging from 20 to 45°C, at pH 7.0. The measurements were performed at a scattering angle of 173° using a 4mW He-Ne laser operating at 632.8 nm. The hydrodynamic diameter and polydispersity were recorded from the correlation function curve and light scattering intensity values.

## 3. Results and discussion

### 3.1 *H*. *volcanii* S-layer protein extraction

**Fig 1:**
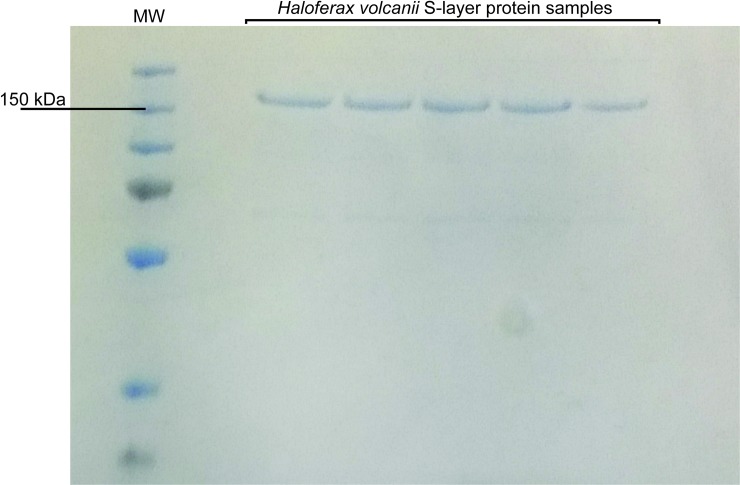
SDS-PAGE electrophoretic migration pattern of the *H*. *volcanii* S-layer protein.

S-layer protein samples were obtained as previously described in the literature [31]. As already mentioned, the *H*. *volcanii* S-layer protein theoretical molecular weight is of approximately 85.2 kDa. However, this value is notoriously lower than the apparent molecular mass observed in SDS-PAGE analyses (Fig 1). Nonetheless, this result has been previously reported in other studies [31] and the same anomalous electrophoretic behavior has been described for the *Halobacterium salinarum* S-layer protein [39]. Both proteins share remarkable similarities in their primary structure and hydrophobicity profiles [31, 39]. It has been suggested that these proteins may have reduced SDS binding capacity due to their high amount of hydrophilic residues, causing reduced electrophoretic mobility, leading to molecular weight overestimations [31].

### 3.2 Structural influences of pH on the *H*. *volcanii* S-layer protein

**Fig 2:**
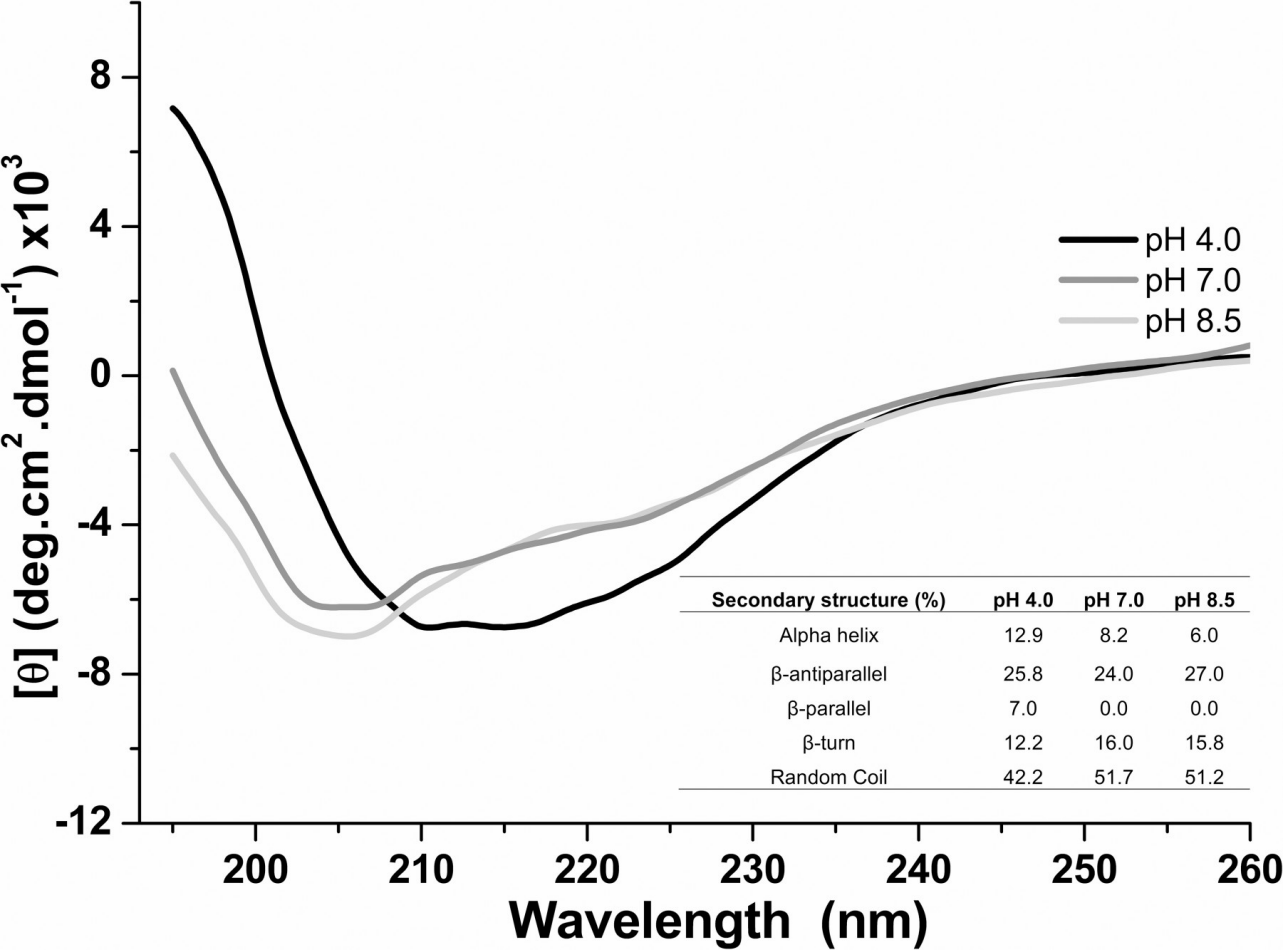
Far-UV CD spectra of the *Haloferax volcanii* S-layer protein at pH 4.0, 7.0 and 8.5, at 25°C.

The effect of pH on the *H*. *volcanii* S-layer protein secondary structure was evaluated by circular dichroism. The Far-UV CD spectra (190–260 nm) were recorded at pH 4.0, 7.0 and 8.5, 25°C (Fig 2). At pH 4.0, a predominance of parallel/antiparallel/turn β-sheets (45.0%) and random coil (42.2%) structures were estimated, followed by α-helices (12.9%). In contrast, at pH 7.0 a decrease of α-helices (12.9% to 8.2%) and parallel/antiparallel/turn β-sheets (45.0% to 40.0%) was observed, with an increase of random coils (42.2% to 51.7%). Interestingly, at pH 8.5 a predominance of parallel/antiparallel/turn β-sheets (42.8%) and random coil (51.2%) structures occurred and an expressive reduction of α-helix contents (6.0%) were estimated when compared to the results obtained at pH 4.0. These results indicate pH dependent secondary structure conformational changes on the *H*. *volcanii* S-layer protein, which becomes more disordered as pH increases.

It is worth highlighting that high β-sheet contents were detected in all pHs analyzed. To date there have been very few studies characterizing S-layer protein secondary structures in *Archaea*. Considering the lack of available structural models and the low comparability between archaeal S-layer protein gene and amino acid sequences, it is difficult to draw a definite conclusion with regard to the existence of global structural similarities in this domain of life [40]. Despite this, high amounts of β-sheet structures were detected in the S-layer proteins from methanogens such as *Methanothermus fervidus*, *Methanothermus sociabilis* and *Methanosarcina acetivorans* [30, 41]. Considering that β-sheets have been described as a fundamental structural factor in establishing intermolecular and intramolecular interactions in proteins [42], it has been suggested that such structures may be involved in the interactions between S-layer protein units [41]. Indeed, similar results were also obtained in studies describing the *Staphylothermus marinus* S-layer morphological unit, the tetrabrachion, where high β-sheet amounts were detected at the interface between monomeric units [43]. If the *H*. *volcanii* S-layer lattice and self-assembly properties are also influenced by these structures, it is possible that the protein β-sheets might be concentrated at intermolecular contact regions. Thus, considering that circular dichroism assays revealed secondary structure changes as a function of pH, it would be likely that such a factor influences the *H*. *volcanii* S-layer lattice and self-assembly properties. It is worth mentioning that the S-layer protein theoretical isoelectric point is 3.44 and thus carries a net negative charge at higher values, which could lead to the structural changes reported here.

**Fig 3:**
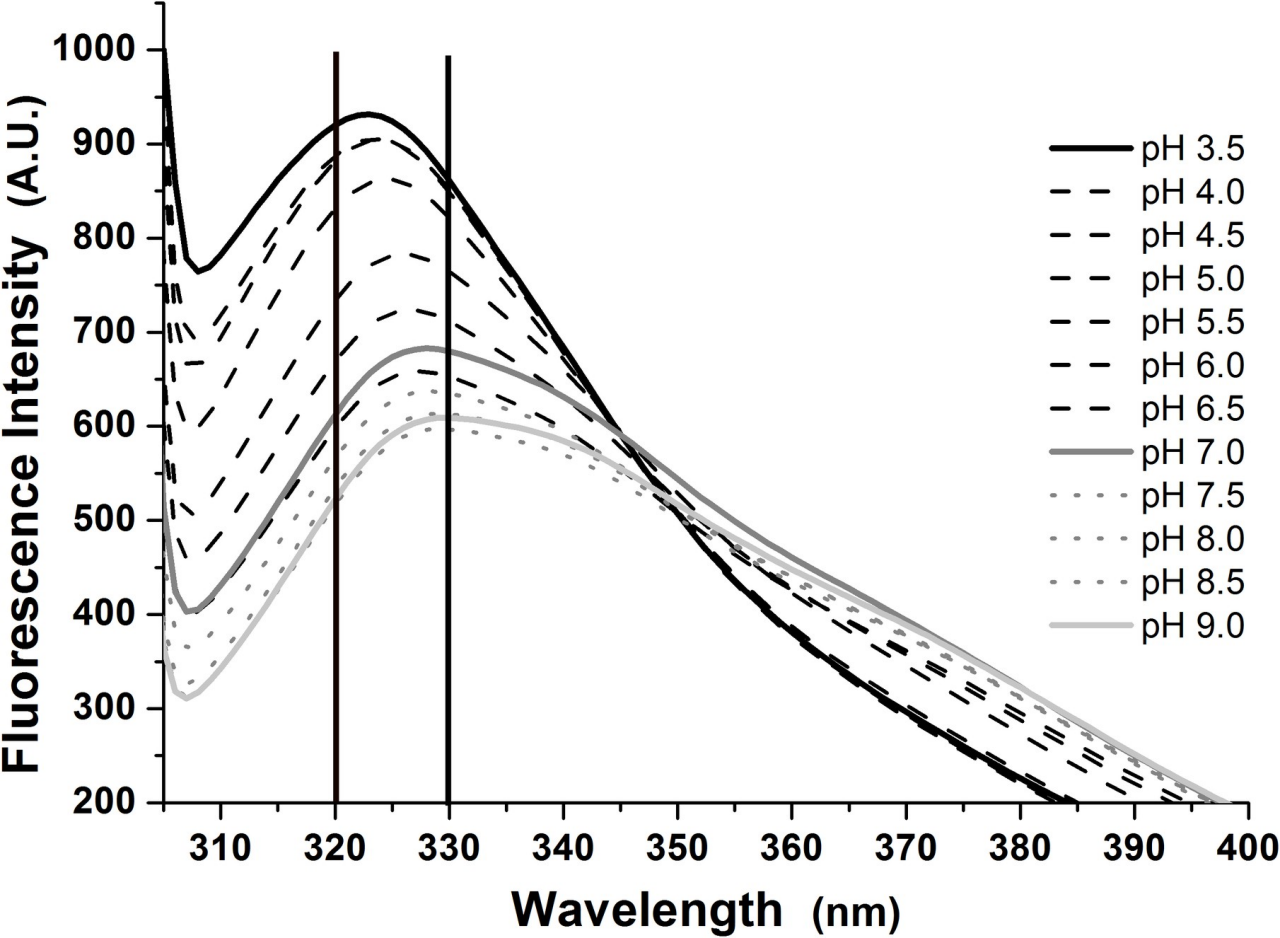
Fluorescence spectra of the *Haloferax volcanii* S-layer protein at 25°C and pH values ranging from 3.5 to 9.0.

Fluorescence spectroscopy was used to evaluate the protein tertiary structure under different pHs (Fig 3). The spectra obtained in values between 3.5 and 9.0 exhibited a red shift from 322 to 332 nm, at values ranging from acidic to neutral, indicating solvent exposed tryptophan residues as a consequence of protein conformational changes due to charged amino acid side chain ionization and non-covalent interaction rearrangements as a function of pH. The *H*. *volcanii* S-layer protein contains 178 amino acid residues that become charged at higher pH values, leading to side chain structural rearrangements. Additionally, a more pronounced red shift from 340 to 350 nm was observed at higher pH values, indicating that one of the protein’s three Trp residues became exposed to the solvent, while the others remained buried. This result indicates partial unfolding of the S-layer protein with the increase in pH. Interestingly, maximum thermal stability occurred at both acidic and neutral conditions (Section 3.3). Together, these results indicate conformational changes and partial unfolding of the S-layer protein as pH becomes higher.

With the increase in pH, the emission spectra also exhibited a decrease in intensity. A plethora of factors can cause this effect, such as proton transfer between charged residues, electron transfer caused by peptide bonds on the protein’s core, resonance energy transfer between tryptophan residues, and interactions between the solvent and exposed tryptophan residues, among others [44–46]. Therefore, both the increase in emission wavelength and decrease in intensity indicate conformational changes in the *H*. *volcanii* S-layer protein structure, which leads to higher exposure of tryptophan residues with the increase in pH.

### 3.3 *H*. *volcanii* S-layer protein structural stability

**Fig 4:**
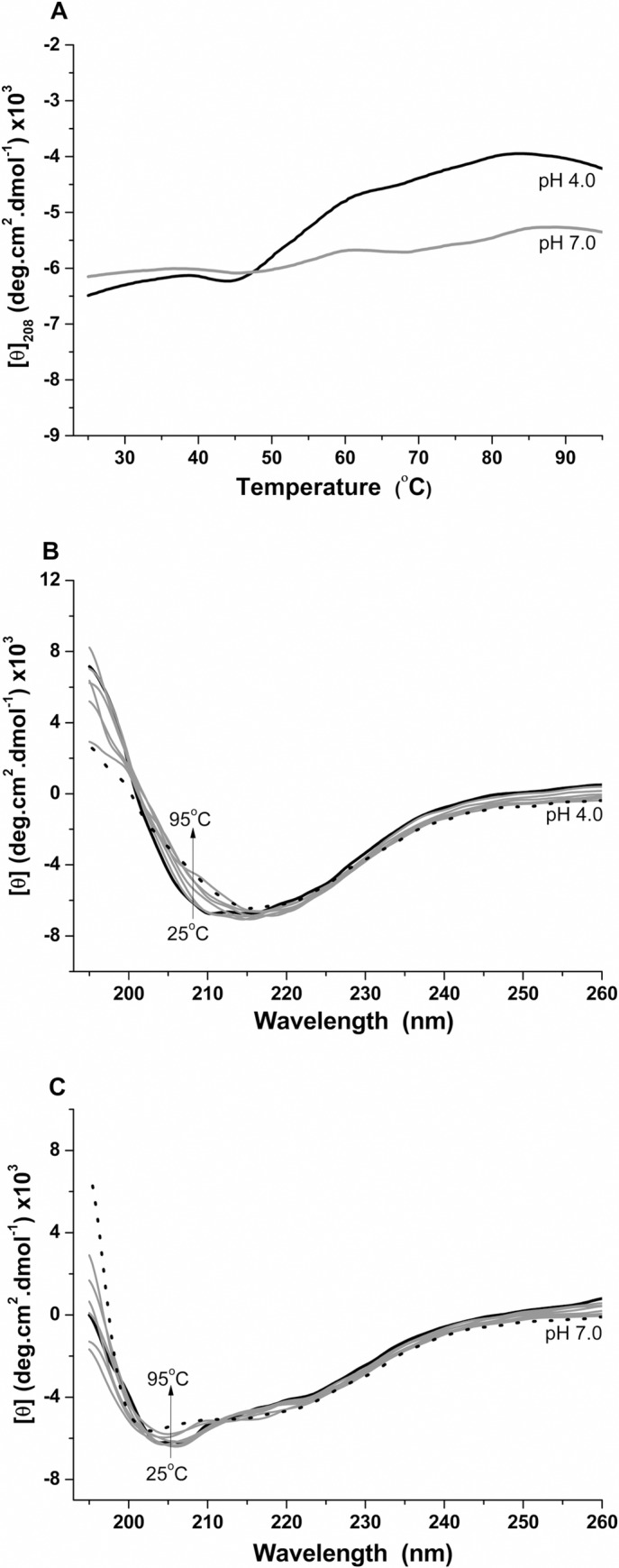
Thermal stability assays performed through circular dichroism on the *Haloferax volcanii* S-layer protein at temperatures varying from 25 to 95°C. A) Unfolding curves recorded at pH 4.0 and 7.0; B) Far-UV CD spectra at pH 4.0; C) Far-UV CD spectra at pH 7.0.

The structural stability of the *H*. *volcanii* S-layer protein was evaluated through circular dichroism at temperatures varying from 25 to 95°C. Thermal denaturation curves were obtained considering the values of [θ]_208nm_ as a function of temperature at acidic (4.0) and neutral (7.0) pHs (Fig 4A) and similar molar ellipticity values were observed. Additionally, the thermal denaturation curve obtained at pH 7.0 displayed a slight increase in dichroic signal until 40°C and then remained constant. These results suggest that the *H*. *volcanii* S-layer protein is thermostable at acidic and neutral conditions. The Far-UV CD spectra obtained at pH 4.0 and pH 7.0 are also indicative of the protein’s thermal stability, considering that similar dichroic profiles throughout the temperature range evaluated were detected, with small signal changes at 208 nm (Fig 4B and 4C). Together, these results indicate that the protein retains its folding profile with the increase in temperature, suffering only minor structural changes. It is worth pointing out that *H*. *volcanii* is not a thermophilic organism, with an optimal growth temperature of 45°C [14]. Given this, these results are intriguing in light of an evolutionary perspective when contemplating the events that could have led to such cell envelope structural features in a mesophilic organism. It is believed that the archaeal common ancestors were hyperthermophilic and mesophilic groups then adapted to lower temperatures during archaeal evolution as a consequence of receiving bacterial genes through interdomain horizontal gene transfer [47]. As such, it is possible that this S-layer structural feature is a vestige of the archaeon’s evolutionary history.

The oligomeric form tendency of *H*. *volcanii* S-layer proteins was evaluated at temperatures ranging from 20 to 45°C, pH 7.0, through dynamic light scattering (DLS) measurements. Our results reveal differences in the protein’s oligomeric and aggregate forms with the increase in temperature (Table 1). At temperatures between 20 and 35°C, two distinct monodisperse and polydisperse forms were detected. Monodisperse samples tend to be composed of particles or molecules of uniform shape and size, with higher probability of assembly into stable ordered structural oligomeric complexes [48]. In contrast, polydisperse forms are often associations of different populations of molecules or particles, with higher tendency to aggregation. The monodisperse form represented a higher percentage of total detected mass at all analyzed temperatures. Here, an oligomeric structure formed by approximately 24 monomers (icositetraedric form) was detected at 20°C and an oligomeric structure of approximately 37–41 monomers between 25 and 35°C. However, at 40 and 45°C only one polydisperse form was detected, with an increase in hydrodynamic radius values, suggesting the presence of larger aggregates at higher temperatures. Together, these results indicate that although the *H*. *volcanii* S-layer protein structure is not greatly affected by an increase in temperature at neutral pH values, the kinetics of self-assembly are apparently influenced by this factor.

**Table 1:** Effect of temperature, varying from 20 to 45°C, on the oligomeric forms of the *H*. *volcanii* S-layer protein at pH 7.0 evaluated through dynamic light scattering.

*Temperature (°C)*	*Oligomeric forms*	*Mass (%)*	*Polydispersity(%)*	*Hydrodynamic diameter (nm)*
20	2	1–59.0 2–41.0	1–11.3 (M) 2–28.5 (P)	1–15.5 ± 1.7 2–56.5 ± 16.8
25	2	1–54.6 2–45.4	1–14.9 (M) 2–29.2 (P)	1–18.6 ± 2.8 2–59.2 ± 17.7
30	2	1–54.2 2–45.8	1–14.5 (M) 2–31.3 (P)	1–19.5 ± 2.9 2–59.2 ± 19.4
35	2	1–50.9 2–49.1	1–11.2 (M) 2–25.1 (P)	1–18.6 ± 2.1 2–56.5 ± 14.6
40	1	100.0	32.5 (P)	62.0 ± 18.8
45	1	100.0	26.0 (P)	62.0 ± 16.3

M: monodisperse; P: polydisperse

### 3.4. Influences of salinity on the *H*. *volcanii* S-layer protein structure and self-assembly

**Fig 5:**
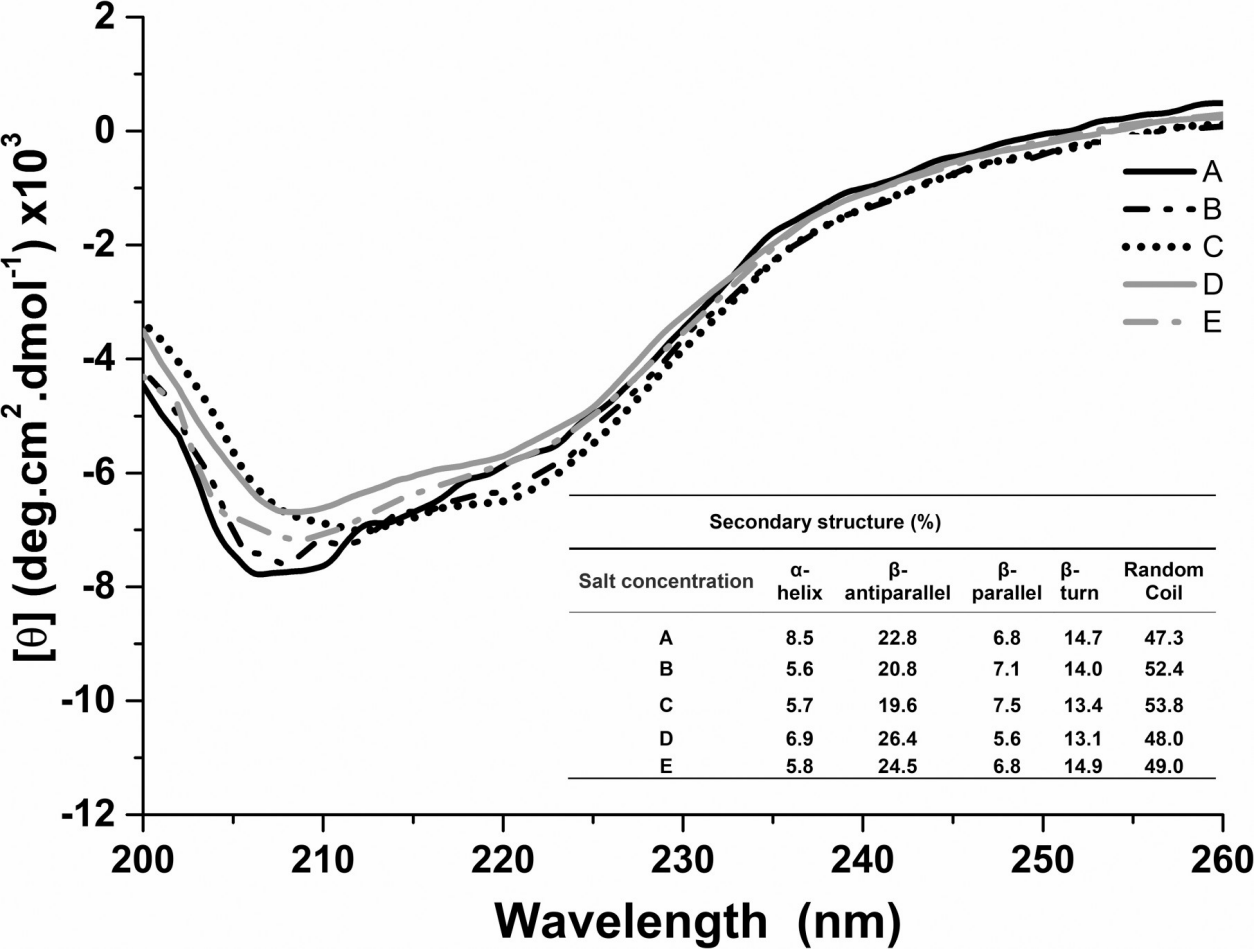
Far-UV CD spectra of the *Haloferax volcanii* S-layer protein at 25°C, pH 6.8, at different salt concentrations. A: 0.001 M CaCl_2_; B: 0.01 M CaCl_2_; C: 2.14 M NaCl and 0.01 M CaCl_2_; D: 0.25 M MgCl_2_; E: 2.14 M NaCl and 0.25 M MgCl_2_.

As previously mentioned, *H*. *volcanii* cells are halophilic and the S-layer is the only cell wall component described for this archaeon, in direct contact with the environment. Therefore, it seems likely that the salinity of the environment influences the S-layer protein structure. We investigated this issue through fluorescence spectroscopy and circular dichroism. Far-UV CD spectra at pH 6.8 were obtained at the following salt concentrations: 0.001 M CaCl_2_; 0.01 M CaCl_2_; 2.14 M NaCl and 0.01 M CaCl_2_; 2.14 M NaCl and 0.25 M MgCl_2_; 0.25 M MgCl_2_. These conditions were the same as those employed in previous studies evaluating the *H*. *volcanii* cell envelope [22] and similar to the composition of most growth media used for this organism [49]. The obtained spectra exhibited slight changes in dichroic signal intensities close to 218–222 nm and 205 nm, depending on the salt solution used. In addition, the adjusted spectra also indicated slight changes in the protein’s secondary structure content, according to these same conditions (Fig 5).

In the presence of 0.001 M CaCl_2_, 8.5% of α-helix and 44.3% of parallel/antiparallel/turn β-sheet contents were estimated. However, when the CaCl_2_ concentration was increased to 0.01 M, a decrease in α-helix content (5.6%), maintenance of β-sheets (41.9%) and an increase in unordered structures (47.3% to 52.4%) was detected. Similarly, in the presence of both 2.14 M NaCl and 0.01 M CaCl_2_, 40.5% of β-sheets were estimated, as well as low amounts of α-helices (5.7%). However, the results using 0.25 M MgCl_2_ in the absence and presence of 2.14 M NaCl indicated a decrease in α-helices (8.2% to 6.9–5.8%) and an increase in β-sheets (40.0% to 45.1–46.2%) (Fig 5) when compared to the estimations obtained at pH 7.0 without the presence of Mg^2+^ ions (Fig 2). It is known that bivalent cations commonly bind to S-layer proteins [50] and it is likely that the Ca^2+^ and Mg^2+^ ions formed interact with the *H*. *volcanii* S-layer protein, affecting its structure. These changes in structure could likely influence the protein’s self-assembly properties. Interestingly, it has been reported that the bacterial S-layer protein SbpA requires Ca^2+^ ions for self-assembly [51], indicating that these ions might be necessary for lattice formation in different phylogenetic groups. As previously discussed, high β-sheet amounts have been detected in S-layer proteins of other archaea [30, 41, 43], and it has been hypothesized that such structures are important in intermolecular protein interactions [41]. Considering that studies investigating bacterial S-layer proteins also revealed high β-sheet contents [20, 52], it seems likely that this factor indeed influences functional properties of these proteins.

**Fig 6:**
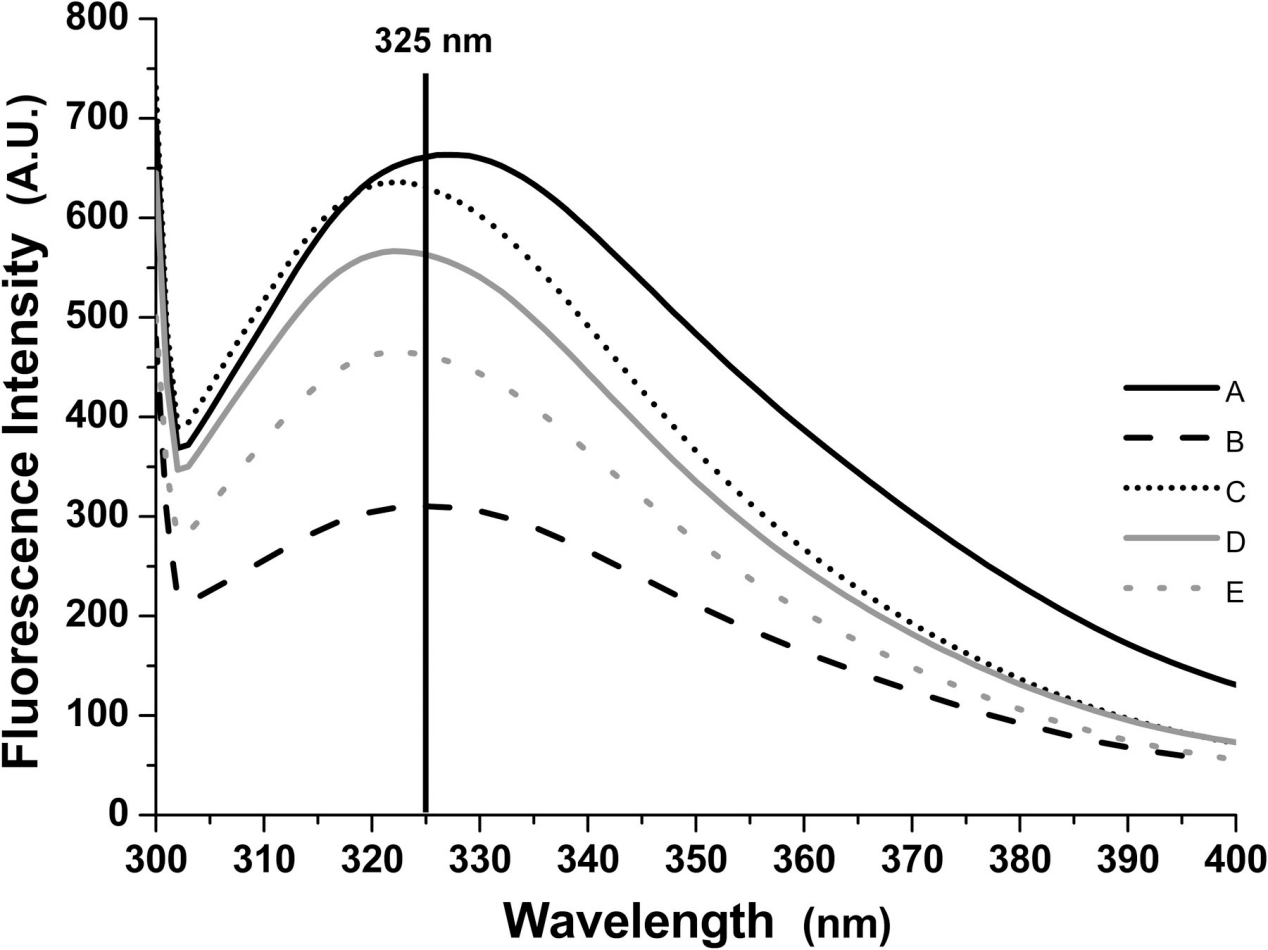
Fluorescence emission spectra of the *Haloferax volcanii* S-layer protein at 25°C, pH 6.8, in different salt concentrations. A: 0.001 M CaCl_2_; B: 0.01 M CaCl_2_; C: 2.14 M NaCl and 0.01 M CaCl_2_; D: 0.25 M MgCl_2_; E: 2.14 M NaCl and 0.25 M MgCl_2_.

Fluorescence spectroscopy was also performed to evaluate changes in the *H*. *volcanii* S-layer protein tertiary structure under the same conditions (Fig 6). When comparing the spectra obtained with CaCl_2_ 0.001 and 0.01 M, a decrease in emission wavelength from 327 to 325 nm was detected, as well as a decrease in emission intensity. This result indicates that the Ca^2+^ ions formed interact with the protein’s side chains, resulting in changes in fluoresce emission. In the presence of both 2.14 M NaCl and 0.01 M CaCl_2,_ the decrease in emission wavelength becomes more evident, reaching a value of 322 nm and with increases in emission intensity. This result indicates that the addition of NaCl and higher CaCl_2_ concentrations leads to reduced exposure of the protein’s tryptophan residues to the environment. Interestingly, while in the presence of 2.14 M NaCl, alteration from 0.01 M CaCl_2_ to 0.25 M MgCl_2_ causes a decrease in emission intensity and an increase in wavelength. This change therefore apparently causes higher exposure of the protein’s tryptophan residues to the environment. However, when incubated with 0.25 M MgCl_2_, the addition of 2.14 M NaCl caused a decrease in emission wavelength and intensity, similar to the results obtained with 0.01 M CaCl_2_. Thus, in both cases the addition of 2.14 M NaCl caused reduced exposure of the protein’s tryptophan residues. As *H*. *volcanii* suffer osmotic lysis in environments with low NaCl concentrations, the results obtained in the presence of this salt may therefore be more representative of the protein structure when attached to the cell surface in nature.

**Fig 7:**
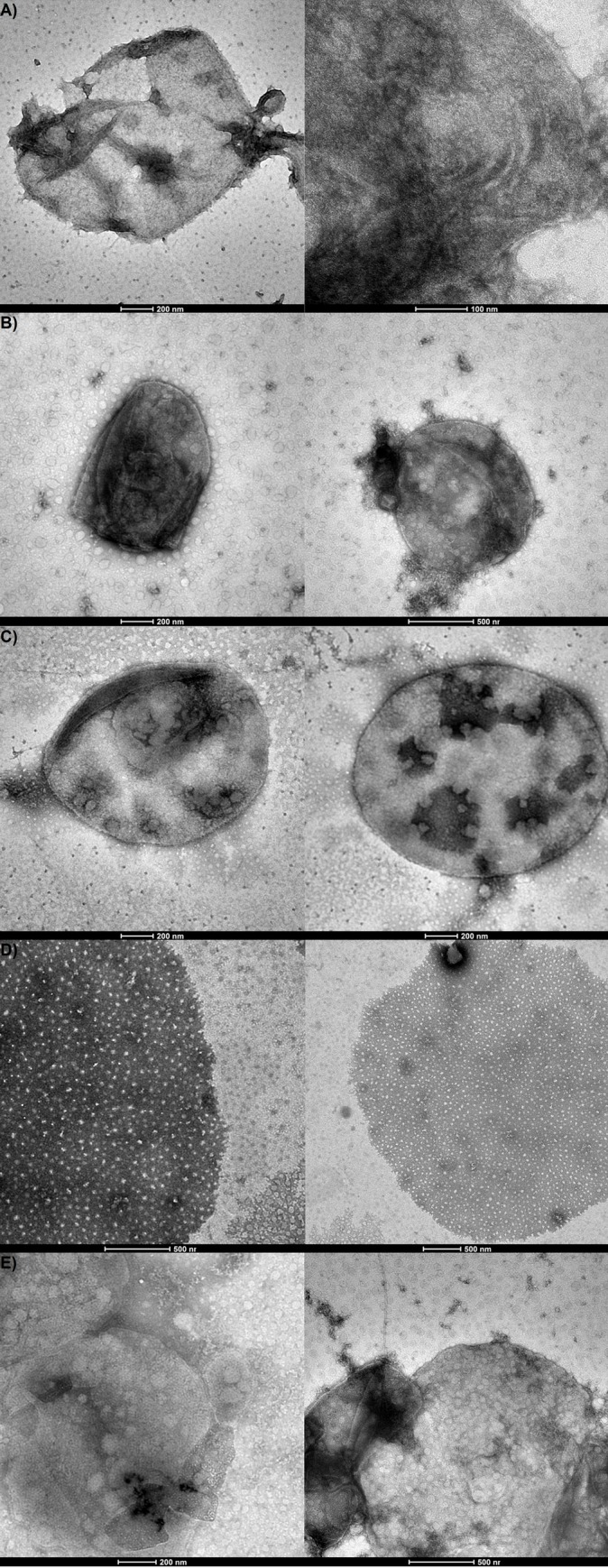
Transmission electron microscopy images of *Haloferax volcanii* cell envelope preparations at different salt concentrations. A: 0.001 M CaCl_2_; B: 0.01 M CaCl_2_; C: 2.14 M NaCl and 0.01 M CaCl_2_; D: 0.25 M MgCl_2_; E: 2.14 M NaCl and 0.25 M MgCl_2_.

Considering that the results obtained through circular dichroism and fluorescence spectroscopy indicated salinity as a factor that influences the S-layer protein structure, *H*. *volcanii* cell envelope preparations were performed at the same salt concentrations previously employed. Transmission electron microscopy images obtained through negative staining revealed notorious differences among the cell envelope preparations under different conditions, and similar to those described in previous reports [21, 22] (Fig 7). It is worth pointing out that in the presence of only bivalent cations, *H*. *volcanii* whole cells suffer osmotic lysis, releasing cell debris and vesicles [22, 53]. However, cell envelope preparations under these conditions were shown to exhibit preserved cell morphology (Fig 7A, 7B and 7D). Cell envelope preparations incubated with 0.001 M CaCl_2_ (Fig 7A) were more fragile than those incubated at 0.01 M (Fig 7B), where round forms were more frequent. When incubated with 2.14 M NaCl and 0.01 M CaCl_2_, cell envelopes had a distinct slightly bloated rounded shape (Fig 7C), an aspect previously described in the literature [22]. When in the presence of only 0.25 M MgCl_2_, cell envelopes displayed a fragile aspect, with breaches throughout the envelope surface (Fig 7D). However, when incubated with both 2.14 M NaCl and 0.25 M MgCl_2_ (Fig 7E), round shaped cell envelopes were again observed.

Together, these results strongly indicate that salinity is a determining factor in S-layer properties in *H*. *volcanii*. The circular dichroism and fluorescence spectroscopy results detected changes in the S-layer protein’s secondary and tertiary structures according to the environment’s salinity, and cell envelope preparations displayed different aspects when under different conditions. These differences are likely related to structural changes in the protein according to ionic concentrations and valence in the environment. It has been shown that salt concentrations influence the *N*-glycosylation process in the protein, with changes concerning both glycan and glycosylation sites in response to salinity variation [27, 54]. Furthermore, this post-translational modification is also related to survival in hypersaline environments [27]. Thus, the salinity of the environment likely affects the *H*. *volcanii* S-layer protein both in structural and post-translational aspects. Perhaps this susceptibility to external factors of salinity and pH is related to the S-layer protein being in constant contact with hypersaline environments.

The S-layer protein of *Halobacterium salinarum*, an extreme haloarchaeon, displays similarities to *H*. *volcanii* [55]. For this organism’s S-layer, the expected lattice pattern in membrane preparations has been observed at 5 M NaCl, but not at lower concentrations [56–58], indicating a role of salinity in cell envelope stability. Similar results were obtained in the haloarchaeon *Haloarcula japonica* S-layer, with stability dependent on the environment’s salt conditions [59]. Furthermore, studies have shown that *N*-glycosylation and sulfation of S-layer glycans in *Halohasta litchfieldiae* and *Halorubrum lacusprofundi*, two cold-adapted haloarchaea, are regulated by the temperature of the environment [60]. Thus, salinity and other environmental conditions display influences on haloarchaeal S-layer protein post-translational modifications, lattice and structure, indicating that this feature might be a common denominator among organisms from this group. However, the lack of detailed structural studies on these proteins hinders comparisons that can be made between the S-layers across these organisms.

## 4. Concluding remarks

To the best of our knowledge, *H*. *volcanii* S-layer protein structure and behavior under different environmental conditions are described here for the first time. Our results indicate that the protein is stable at high temperatures in acidic and neutral pHs and that this factor, as well the environment salt concentration, affects the protein’s secondary and tertiary structure. Furthermore, micrograph images of cell envelope preparations revealed notorious differences at different salt concentrations, reinforcing the idea that salinity influences the S-layer protein structure and functional properties. Changes have been reported concerning the protein’s post-translational modifications according to environmental conditions in both *H*. *volcanii* and other haloarchaea. Moreover, a dependence on salinity for S-layer structural stability has been commonly reported in other halophilic organisms. Thus, it can be argued that haloarchaeal S-layer proteins are susceptible to external factors.

Our study also revealed high β-sheet contents on the *H*. *volcanii* S-layer protein. As previously mentioned, such structures have been commonly detected on S-layer proteins of both bacteria and archaea [20], despite the low amino acid sequence homology found between different phylogenetic groups. An acidic isoelectric point is another common S-layer protein feature, though higher values have been found on *Methanothermus fervidus* [41] and some lactobacilli [61]. When amino acid sequence identity can be detected, these are usually higher on the N-terminal region when compared to the C-terminal portion [20]. Indeed, conserved four to six amino acid sequences were observed in S-layer proteins of different *Bacillus* species [20, 62] and S-layer homologous (SLH) motifs have been described at the N-terminal portion in several gram-positive bacteria [63–66]. However, such SLH motifs have not been detected on *Geobacillus stearothermophilus* wild strains [62] and *Lactobacillus* spp. [67]. To date, the most detailed S-layer structural studies were performed using gram-positive bacteria [68], with fewer studies focusing on gram-negative and archaeal S-layers. Although a few features appear to be common, homology among S-layers of phylogenetically distant organisms is low, hindering global comparison analyses.

While there is significant interest in S-layer proteins overall, there is a lack of structural information available in the literature. There are several reasons for this, such as the fact that S-layer proteins tend to display high molecular mass (ranging from 40 to 200 kDa) [20] and usually undergo post-translational modifications, hindering analyses through nuclear magnetic resonance methods. Furthermore, S-layers have a tendency of forming two-dimensional lattices and do not usually form the three-dimensional crystals necessary for structure determining methods such as X-ray crystallography [50]. All the same, future studies are likely to enlighten our knowledge on archaeal S-layer proteins, advancing our understanding of their structural properties as well as their roles in the organism’s physiology.
